# Effect of Adjuvant Electroconvulsive Therapy Compared to Antipsychotic Medication Alone on the Brain Metabolites of Patients with Chronic Schizophrenia: A Proton Magnetic Resonance Spectroscopy Study 

**Published:** 2018-07

**Authors:** Mehrzad Lotfi, Mehdi Ghaderian Jahromi, Ali Firoozabadi, Leila Razeghian Jahromi

**Affiliations:** 1 Department of Radiology, School of Medicine, Medical Imaging Research Center, Shiraz University of Medicine Sciences, Shiraz, Iran.; 2 Department of Psychiatry, School of Medicine, Shiraz University of Medicine Sciences, Shiraz, Iran.

**Keywords:** *Brain Metabolites*, *Electroconvulsive Therapy*, *Magnetic Resonance Spectroscopy*, *Schizophrenia*

## Abstract

**Objective:** Schizophrenia is a common psychiatric disease and is characterized by changes in several brain metabolites detectable by magnetic resonance spectroscopy (MRS). Electroconvulsive therapy (ECT) is a general method of management for most severe psychiatric conditions that may play a role in changing the brain metabolites. This study examined the effectiveness of adjuvant ECT with oral medication compared to that of oral second generation antipsychotic medication alone on brain metabolites in patients with chronic schizophrenia.

**Method** **:** This study was conducted on 20 patients with chronic schizophrenia who were admitted to a hospital; of them, 10 underwent ECT as an adjuvant therapy with oral medication at least 8 times, and 10 patients were given a second- generation antipsychotic therapy drug (risperidone and olanzapine) without ECT for at least 4 weeks. MRS was used to assess brain metabolites, including N-acetyl aspartate (NAA), choline (Cho), creatine (Cr), myoinositol (MI), and Glx (glutamate [Glu] and glutamine [Gln]), in the left prefrontal cortex, left thalamus, left hippocampus, and left occipital cortex. Differences between the 2 groups were not significant, except for method of treatment.

**Results: **The NAA/Cr ratio in the left prefrontal cortex was significantly higher in ECT-treated patients (P = 0.035). In addition, the Cho/Cr ratios in the left prefrontal cortex and left thalamus were statisticaly lower in the ECT-treated patients than those treated with oral antipsychotic drugs alone (P = 0.019). No statistically significant changes were observed between the 2 groups in other sites of the brain. In addition, no statistically significant differences were detected between the 2 groups in SAPS and DES scores.

**Conclusion: **Compared to oral antipsychotic drug treatment, ECT had improving effects on at least 2 metabolites in the brains of patients with schizophrenia. Therefore, ECT may have a neuroprotective effect in these patients.

Schizophrenia is a complex psychiatric disorder. It is characterized by abnormal associations, autistic behavior and thinking, abnormal affects, ambivalence, delusion, hallucinations, and social withdrawal. Risk factors include family history, birth in the winter, blood group incompatibility, and influenza infection during pregnancy. Schizophrenia has an equal prevalence between men and women and most commonly occurs at the ages of 25 and 50 years (1). Electroconvulsive therapy (ECT) plays an important role in the treatment of schizophrenia, improving both positive symptoms (2, 3). 

ECT results in changes in cellular membrane action potential in a specific time, indicating that it is working (1). 

ECT results in decreased glucose consumption and cell metabolism, especially in the frontal lobe, which may have a positive effect, similar to that of treatment response, on oral antipsychotic drug therapy in schizophrenia patients (1, 2, and 3). 

Changes in brain metabolites, including N-acetyl aspartate (NAA), choline (Cho), creatine (Cr), myoinositol (MI), and glutamate (Glu) + glutamine (Gln), in different parts of the brain in these patients after ECT can be evaluated by magnetic resonance spectroscopy (MRS).

MRS is a method of brain imaging similar to magnetic resonance imaging (MRI) that detects the strength of brain metabolites instead of imaging by detecting the spectrum of signals produced. Two metabolite localization methods have been described for MRS: (1) point-resolved spectroscopy (PREES), with a time-to-echo (TE) of 135-288 ms that can be used for major metabolites, such as NAA, Cr, Cho, and lactate (Lac); (2) stimulated echo acquisition (STEAM), with a TE of 20-30 ms that can be used to detect major metabolites as well as minor metabolites, such as MI, Glu, Gln, alanine (Ala), and gamma-aminobutyric acid (GABA) (4,5,6,7,8,9). NAA is the prominent metabolite in adults and is detected at 2.01 ppm by MRS, with a reference chemical shift. NAA is also a reported neuronal component of brain tissue (9, 10, and 11). Cr is an energy-saving metabolite detected at 3.03 ppm (9, 10 and 11). Cho is a metabolite associated with cell membrane turn over that is detected at 3.2 ppm (9, 12). MI is a glial marker detected at 3.56 ppm that affects cellular detoxification (9, 13). 

Glu and Gln are detected at 2.1 and 2.5 ppm and affect cellular induction and neurotransmitter regulation, respectively. These metabolites play roles in epilepsy and schizophrenia (14). In schizophrenia patients, NAA levels are low in the frontal cortex, thalamus, and temporal and anterior cingulate gyrus (10, 11), while Glu and Gln levels are high in the thalamus and anterior cingulate gyrus (14). Cho levels in the basal ganglia and anterior cingulate gyrus are high (12), while MI levels are low in the frontal lobe (13) compared to those in controls. 

A 2012 study reported significantly increased NAA/Cr ratios in the prefrontal cortex and thalamus of 31 schizophrenia patients who had undergone modified ECT for 8 times (15). A 2014 study also reported an increased NAA/Cr ratio in the prefrontal lobe after 6 months of antipsychotic treatment (16). MI and NAA levels were increased in the thalamus of schizophrenia patients treated with new antipsychotic drugs (risperidone) in a study published in 2005 (17). A decreased Glu/Cr ratio in the temporal cortex and increased NAA/Cr ratio in the thalamus were also observed following antipsychotic treatment in schizophrenia patients (18).

Also, antipsychotic treatment had a positive effect on brain metabolite levels in mice (19). However, the severity of symptoms was associated with metabolites. Positive symptoms are directly correlated with Cho/Cr ratio and indirectly correlated with NAA/Cr ratio, while visual inhibition in the visual cortex is indirectly correlated with GABA level (20, 21). A 1997 study reported that the dissociative symptoms in schizophrenia patients are more prominent compared to those in controls and that these symptoms are associated with hallucinations and delusion (22).

A recent large retrospective study done by Kaster TS and his team found that ECT is an effective treatment choice for schizophrenia, which is tolerated by most of the patients (23). Thus, considering the possible role of changes of different metabolites in schizophrenia patients and their changes after various types of treatments (24) and taking into account the lack of data on the effect of ECT on MRS data, we conducted this study using MRS to compare brain metabolites at different sites between schizophrenia patients treated with adjuvant ECT and a second- generation oral antipsychotic drug alone to examine the effectiveness of ECT on improving brain metabolites. Past studies merely focused on the effect of oral medication on brain metabolites. However, in this study, the effect of adjuvant ECT on brain metabolites was evaluated in comparison to oral medication.

## Materials and Methods

This cross- sectional study was conducted on 20 patients with chronic schizophrenia who were admitted in a hospital. Informed consent was obtained from all the participants and their families. In this study, 10 patients who underwent ECT for at least 8 times and at least 2 times weekly and took a second- generation oral antipsychotic medication and 10 patients who took a second- generation oral antipsychotic drug for at least 4 weeks were included. 

Oral medication was risperidone or olanzapine. In each group, five patients received risperidone and five received olanzapine, but the dosage of medication was different among patients. The cases were randomly selected among the admitted patients and included in this study during their routine treatment. However, we did not interfere with their routine treatment. The mean age of the two groups was 37 years and all participants were right-hand dominant. According to some studies, metabolites ratios do not depend on sex (25), but in our study, the number of men and women was equal in the 2 groups. Time of admission was similar in the two groups. Duration of disease in the two groups was equal and was about 8 years. MRS was used to measure levels of brain metabolites after treatment, including NAA, Cho, Cr, MI, and Glx, in the left prefrontal cortex, left thalamus, left hippocampus, and left occipital cortex. Time of each imaging was about 30 minutes and anesthesia was not used for any patient. The levels of these metabolites were then compared and correlated with SAPS and DES scores between the two treatment groups with MRS simultaneously. Differences between the 2 groups were not significant, except for method of treatment.

The Scale for Assessment of Positive Symptoms (SAPS) is a 35-question tool used to evaluate patient symptoms such as hallucinations and delusion (1). The Dissociative Experience Scale (DES) is a 28-question tool used to evaluate dissociative symptoms in patients, including absorption, amnesia, identity attention, depersonalization, and derealization (22).

The criteria for the selection of patients were based on the Diagnostic and Statistical Manual of Mental Disorders, 4th Edition, Text Revision (DSM-IV-TR). Agoraphobia, history of any brain problems, patients’ irritability, and any MRI incompatibility device in patients were some of the exclusion criteria for this study. Ethical considerations were taken into account in our study. This study was performed using a 1.5 T AVANTO SIEMENS MRI machine with single voxel proton MRS method (2*2*2 cm) using a STEAM sequence by chemical shift water suppression (CHESS).

Statistical analyses were performed using PASW Statistics for Windows, Version 18.0 (SPSS, Inc., Chicago, IL, USA). Shapiro-Wilk and Mann-Whitney tests were used to compare quantitative variables.

## Results

Brain MRS in different brain regions of the patients with schizophrenia was used to obtain data on metabolites, then, the metabolite ratios were compared to Cr.

Based on the results of the MRS test and the Man-Whitney and Shapiro-Wilk test, the NAA / cr level in the left frontal lobe, with the mean of 3/37 (p- value = 0.0335), was significantly higher in the ECT patient group. On the other hand, the Cho/cr ratio in the left prefrontal cortex, with the mean of 0.58 (p- value = 0.019) in the ECT group was lower than the drug treatment group. In the ECT patients, the Cho /cr and NAA/cr ratios in the left thalamus, with the mean of 1.78 and 0.60, respectively, were higher and lower than the drug treatment group, but not significant (p- value = 53.0, and p = 0.50, respectively).

**Figure 1:**
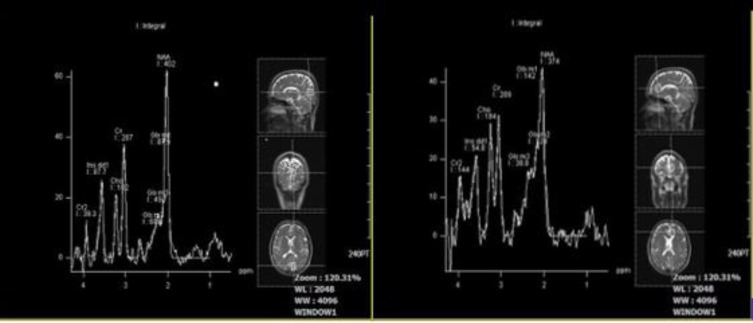
MRS Images Showing Voxel Sites in the Left Prefrontal Cortex, Occipital Cortex, Thalamus, and Hippocampus and Their Associated Spectrums

No significant difference was observed in the other sites of the brain and other metabolites between the 2 groups. Finally, no statistical differences were observed in SAPS and DES scores between the 2 groups, but scores in the ECT-treated group were lower in average than those in the oral treatment group. The mean score for SAPS was 2 in ECT- treated and 2.06 in oral- treated groups, and for DES it was 9.62 in ECT- treated group and 11.1 in the oral- treated patients. In Tables 1, 2 and 3, the mean ratio of brain metabolites in the 2 groups in different sites of the brain was noted. Voxel region of MRS in different sites of the brain is presented in Figure 1.

## Discussion

Due to the popular use of MR scanners and wide range of quantification tools, MRS has become widely used to evaluate psychiatric disorders, including schizophrenia. During the last 3 decades, many studies compared schizophrenia patients and healthy control groups; moreover, treatment studies based on MRS findings were surprisingly comparable in number with cross-sectional investigations (24). Several studies have reported a decrease in NAA/Cr ratios in the prefrontal cortex and thalamus of schizophrenia patients (6, 8, 9 and 10). However, a 2002 study comparing 70 schizophrenia patients and controls reported no definite change in NAA/Cr in the prefrontal cortex and thalamus (26). In a 2005 study of healthy controls (32 men and 40 women), with an average age of 27 years, the NAA/Cr ratio was 2.34 0.37 in the prefrontal cortex and 2.09 0.22 in the thalamus; the Chol/Cr ratio was 1.14 0.15 in the prefrontal cortex and 1.13 0.17 in the thalamus. The ratios did not differ between men and women (25). A number of studies have assessed oral antipsychotic drugs. For example, in 2010, Szulk et al. studied 32 patients with chronic schizophrenia and 21 healthy controls. In their study, 21 patients took risperidone and 11 took olanzapine, and they found that NAA/Cr ratio in thalamus increased in risperidone treated patients (18). Other studies have also observed increased NAA levels in the thalamus and frontal lobe following treatment with risperidone (16, 17). Also, in a study in 2012, it was found that after oral medication glutamate level increased in anterior cingulate lobe with an associated decrease in negative symptoms and an increase in patients’ function (27). In 2008, Bustillo found frontal and striatal NAA reductions in minimally treated patients who showed no change following a 9-month randomized treatment with quetiapine or haloperidol (28).

In addition, a study by Gan et al. in 2012 on 31 chronic schizophrenia patients reported an increased NAA/Cr ratio in the left prefrontal cortex and thalamus in patients who underwent ECT 8 times (15). According to above- mentioned studies, a significant improvement was seen in the brain metabolites of schizophrenia patients after treatment. Also, a recent large retrospective study by Kaster TS et al. supports the effectiveness of electroconvulsive therapy (ECT) as an adjunctive treatment for schizophrenia and shows characteristics related to treatment response (23). Thus, we decided to work on ECT as an adjuvant therapy to evaluate metabolites change.

 Comparison of our results with those of other studies revealed that our findings on the patterns of brain metabolites in schizophrenia patients were similar to those of other studies, meaning that NAA/Cr ratio in target group in prefrontal cortex increased and Cho/Cr ratio in prefrontal and thalamus decreased. However, contrary to other studies on the role of oral antipsychotic therapy, that were mostly based on 4 to 6 weeks oral medication (16, 17,18), our study evaluated the effect of adjuvant ECT on metabolite changes as a complementary method compared to antipsychotic medication alone. Similar to other reference studies, our patients received ECT 8 times (15). Based on our results, ECT may play a significant role in improving brain metabolites and may have a neuroprotective effect on schizophrenia patients.

**Table 1:** Mean Ratios (Cr as Refrence) in 4 Brain Regions (Prefrontal Cortex, Occipital Cortex, Thalamus and Hippocampus) in Electroconvulsive Therapy Treated Patients

**Brain** **region**	**Naa** **pfc**	**Chol** **pfc**	**Is** **pfc**	**Glx** **pfc**	**Naa** **oc**	**Chol** **oc**	**Is** **oc**	**Glx** **oc**	**Naa** **thal**	**Chol** **thal**	**Is** **thal**	**Glx** **thal**	**Naa** **hip**	**Chol** **hip**	**Is** **hip**	**Glx** **hip**
mean	3.376	0.569	0.266	0.777	1.853	0.440	0.334	0.828	1.961	0.607	0.240	0.502	2.648	0.757	0.325	0.635
Standard deviation	0.818	0.196	0.142	0.180	0.491	0.105	0.054	0.133	0.464	0.154	0.206	0.241	0.672	0.204	0.130	0.221

NOTE. pfc = prefrontal cortex; oc = occipital cortex; thal = thalamus; hip = hippocampus

**Table 2:** Mean Ratios (Cr as Reference) in 4 Brain Regions (Prefrontal Cortex, Occipital Cortex, Thalamus and Hippocampus) in Oral Antipsychotic Treated Patients

**Brain** **region**	**Naa** **pfc**	**Chol** **pfc**	**Is** **pfc**	**Glx** **pfc**	**Naa** **oc**	**Chol** **oc**	**Is** **oc**	**Glx** **oc**	**Naa** **thal**	**Chol** **thal**	**Is** **thal**	**Glx** **thal**	**Naa** **hip**	**Chol** **hip**	**Is** **hip**	**Glx** **hip**
mean	2.639	0.800	0.277	0.661	1.788	0.474	0.313	0.723	2.211	0.794	0.264	0.579	2.402	0.890	0.333	0.522
Standard deviation	1.269	0.182	0.096	0.246	0.343	0.078	0.053	0.162	0.265	0.146	0.078	0.274	0.648	0.154	0.124	0.233

NOTE. pfc = prefrontal cortex; oc = occipital cortex; thal = thalamus; hip = hippocampus

**Table 3:** Comparison of Metabolites (Cr as Refrence) Ratios between the 2 Groups (ECT Treated and Oral Medication Treated Alone)

**Brain** **region**	**Naa** **pfc**	**Chol** **pfc**	**Is** **pfc**	**Glx** **pfc**	**Naa** **oc**	**Chol** **oc**	**Is** **oc**	**Glx** **oc**	**Naa** **thal**	**Chol** **thal**	**Is** **thal**	**Glx** **thal**	**Naa** **hip**	**Chol** **hip**	**Is** **hip**	**Glx** **hip**
Mean diff	-2.11	-2.34	-0.20	-1.38	-0.16	-1.22	-1.27	-1.38	-1.93	-2.34	-1.32	-0.69	-1.02	-1.43	-0.41	-1.14
Crit Diff	0.034	0.019	0.838	0.165	0.870	0.220	0.204	0.165	0.054	0.019	0.185	0.487	0.307	0.151	0.677	0.253
P value	0.035^a^	0.019 ^a^	0.842^a^	0.182^a^	0.905^a^	0.243^a^	0.211^a^	0.182^a^	0.052^a^	0.019^a^	0.190^a^	0.497^a^	0.315^a^	0.165^a^	0.684^a^	0.278^a^

NOTE. pfc = prefrontal cortex; oc = occipital cortex; thal = thalamus; hip = hippocampus

## Limitation

In this study, a relatively small sample size, varying disease durations, absent medication dosage, lack of healthy controls, and using 1.5 and not 3 Tesla MR scanner should be mentioned as limitation factors. Thus, additional studies with more patients and healthy controls and considering other mentioned limitations should be conducted to validate the findings of the present study.

## Conclusion

Adjuvant ECT-treated schizophrenia patients had a higher NAA/Cr ratio in the left prefrontal cortex and lower Chol/Cr ratios in the left prefrontal and left thalamus compared to patients who took oral antipsychotic drugs alone. Therefore, ECT may be a method for the treatment and improvement of brain metabolites in these patients.

## References

[1] Carol A T, Sadock BJ, Sadock VA (2009). Schizophrenia and other psychotic disorders: Introduction and overview. Kaplan & Sadock's Comprehensive Textbook of Psychiatry.

[2] Tharyan P, Adams CE (2005). Electroconvulsive therapy for schizophrenia. Cochrane Database Syst Rev.

[3] Small IF, Milstein V, Miller MJ, Malloy FW, Small JG (1986). Electroconvulsive treatment indications, benefits, and limitations. Am J Psychother.

[4] Young K, Maudsley AA (2000). Proton NMR chemical shifts and coupling constants for brain metabolites. NMR Biomed.

[5] Meyerhoff DJ, MacKay S, Bachman L, Poole N, Dillon WP, Weiner MW, Fein G (1993). Reduced brain N-acetylaspartate suggests neuronal loss in cognitively impaired human immunodeficiency virus-seropositive individuals: in vivo 1H magnetic resonance spectroscopic imaging. Neurology.

[6] Lyoo IK, Renshow RF (2002). Magnetic resonance spectroscopy: current and future applications in psychiatric research. Biol Psychiatry.

[7] Malhi GS, Valenzuela M, Wen W, Sachdev P (2002). Magnetic resonance spectroscopy and its applications in psychiatry. Aust N Z J Psychiatry.

[8] Vance AL, Velakoulis D, Marruff P, Wood SJ, Desmound P, Pantelis C (2000). Magnetic resonance spectroscopy and schizophrenia:What have we learnt?. Aust N Z J Psychiatry.

[9] John RH, Daniel TB, Gan JL, Li XQ, Duan HF, Yang JM, Wang CH, Yang XS (2012). Brain proton mangnetic resonance spectroscopy. CT and MRI of whole body.

[10] Basoqlu C, Cetin M, Oner O, Ebrinc S, Semiz UB, Kandilcioqlu H (2006). Comparison of right thalamus and temporal cortex metabolite levels of drug –naïve first-episode psychoyic and chronic schizophreniain patients. Turk Psikiyatri Derg.

[11] Jia YB, Wang Y, Ling XY, Zhong SM, Huang L (2012). Proton magnetic resonance spectroscopy and deffusion tensor imaging study of first episodepatients with positive symptoms os schizophrenia. Nan Fang Yi Ke Da Xue Xue Bao.

[12] Tandon N, Bolo NR, Sanghavi K, Mathew IT, Francis AN, Stanley JA (2013). Brain metabolite alterations in young adults at familial high risk for schizophrenia using magnetic resonance spectroscopy. Schizophr Res.

[13] Kim H1, McGrath BM, Silverstone PH (2005). A review of possible relevance of inositol and the phosphatidylinositol second messenger system (PI – cycle) to psychiatric disorders- focous on magnetic resonance spectroscopy (MRS).studies. Hum Psychopharmacol.

[14] Théberge J, Bartha R, Drost DJ, Menon RS, Malla A, Takhar J (2002). Glutamate and glutamine measuredwith 4.0 Tproton MRS in never –treated patients with schizophrenia and healthy volunteers. Am J Psychiatry.

[15] Gan JL, Li XQ, Duan HF, Yang JM, Wang CH, Yang XS (2012). Proton magnetic resonance spectroscopy study of prefrontal lobe and thalamus in schizophrenia on modified electroconvulsive therapy. Zhonghua Yi Xue Za Zhi.

[16] Grošić V, Folnegović Grošić P, Kalember P, Bajs Janović M, Radoš M, Mihanović M (2014). The effectof atypical antipsychotics on brain N-acetylaspartate levels in antipsychotic –naïve first-episode patients with schizopherenia:a preliminary study. Neuropsychiatr Dis Treat.

[17] Szulc A, Galinska B, Tarasow E, Dzienis W, Kubas B, Konarzewska B (2005). The effect of risperidone on metebolite measures in the frontal lobe,temporal lobe,and thalamus in schizophrenic patients.A proton magnetic resonance spectroscopy(1H MRS). Pharmacopsychiatry.

[18] Szulc A1, Galińiska B, Tarasów E, Dzienis W, Kubas B, Konarzewska B (2010). The influence of atypical anti-psychotic on brain functioning in schizophrenia. A proton magnetic resonance study. Psychiatr Pol.

[19] Harte MK, Bachus SB, Reynolds GP (2005). Increased N-acetylaspartate in rat stiatum following long termadministration of haloperidol. Schizophr Res.

[20] He ZL, Deng W, Li ML, Chen ZF, Collier DA, Ma X (2012). Detection of metabolites in the white matterof frontal lobesand hippocampuswith proton in first-episode treatment naïve-schizophrenia patients. Early Interv Psychiatry.

[21] Tayoshi S, Nakataki M, Sumitani S, Taniguchi K, Shibuya-Tayoshi S, Numata S (2010). GABA concentration in schizophrenia patients and the effects of antipsychotic medication: a proton magnetic resonance spectroscopy study. Schizophr Res.

[22] Spitzer C, Haug HJ, Freyberger HJ (1997). Dissociative symptoms in schizophrenic patients with positive and negative symptoms. Psychopathology.

[23] Kaster TS, Daskalakis ZJ, Blumberger DM (2017). Clinical effectiveness and cognitive impact of electroconvulsive therapy for schizophrenia: a large retrospective study. J Clin Psychiatry.

[24] Bustillo JR (2013). Use of proton magnetic resonance spectroscopy in the treatment of psychiatric disorders: a critical update. Dialogues Clin Neurosci. Dialogues Clin Neurosci.

[25] Safriel Y, Pol-Rodriguez M, Novotny EJ, Rothman DL, Fulbright RK (2005). Refrence values for long Echo Time MR Spectroscopy in healty adult. AJNR AmJ Neuroradiol.

[26] Delamillieure P, Constans JM, Fernandez J, Brazo P, Benali K, Courthéoux P (2002). Proton Magnetic Spectroscopy (H-MRS) in schizophrenia :investigation of right and left hippocampus, thalamus and prefrontal cortex. Schizophr Bull.

[27] Egerton A, Brugger S, Raffin M, Barker GJ, Lythgoe DJ, McGuire PK (2012). Anterior cingulate glutamate level related to clinical status following treatment in first episode schizophrenia. Neuropsychopharmacology.

[28] Bustillo JR1, Rowland LM, Jung R, Brooks WM, Qualls C, Hammond R (2008). Proton magnetic resonance spectroscopy during initial treatment with antipsychotic medication in schizophrenia. Neuropsychopharmacology.

